# Asymmetric Presynaptic Depletion of Dopamine Neurons in a *Drosophila* Model of Parkinson’s Disease

**DOI:** 10.3390/ijms24108585

**Published:** 2023-05-11

**Authors:** Jiajun Zhang, Lucie Lentz, Jens Goldammer, Jessica Iliescu, Jun Tanimura, Thomas Dieter Riemensperger

**Affiliations:** 1 Institute of Zoology, Experimental Morphology and Neuroanatomy, University of Cologne, Zuelpicher Str. 47b, 50674 Cologne, Germany; 2 Neuronal Circuit Division, Institute of Molecular and Cellular Biosciences, The University of Tokyo, 1-1-1 Yayoi, Bunkyo-ku, Tokyo 113-0032, Japan

**Keywords:** Parkinson’s disease, dopamine, neuron degeneration with lateral predominance, *Drosophila*

## Abstract

Parkinson’s disease (PD) often displays a strong unilateral predominance in arising symptoms. PD is correlated with dopamine neuron (DAN) degeneration in the substantia nigra pars compacta (SNPC), and in many patients, DANs appear to be affected more severely on one hemisphere than the other. The reason for this asymmetric onset is far from being understood. *Drosophila melanogaster* has proven its merit to model molecular and cellular aspects of the development of PD. However, the cellular hallmark of the asymmetric degeneration of DANs in PD has not yet been described in *Drosophila*. We ectopically express human α-synuclein (hα-syn) together with presynaptically targeted syt::HA in single DANs that innervate the Antler (ATL), a symmetric neuropil located in the dorsomedial protocerebrum. We find that expression of hα-syn in DANs innervating the ATL yields asymmetric depletion of synaptic connectivity. Our study represents the first example of unilateral predominance in an invertebrate model of PD and will pave the way to the investigation of unilateral predominance in the development of neurodegenerative diseases in the genetically versatile invertebrate model *Drosophila*.

## 1. Introduction

Parkinson’s disease (PD) is the most frequent degenerative movement disorder [1,2,3,4], which is characterized by the pathophysiological degeneration and loss of dopamine neurons (DANs) in the substantia nigra pars compacta (SNPC) of the human brain. This loss of DANs typically yields motor symptoms such as low frequent resting tremors, postural instability, bradykinesia, and rigidity, but PD can also be associated with non-motor symptoms such as depression, anxiety, and dementia (summarized in [5]). The onset of motor symptoms in PD patients is predominantly asymmetric, and the unilateral predominance of motor disorder in the early course of the disease serves as a criterium for a positive diagnosis of PD [6,7,8]. Such asymmetric predominance often persists during the progression of DAN degeneration [9]. Recently, Goelman and colleagues demonstrated that PD patients suffer from strong asymmetrical disruptions of the communication circuitry between the insula and the sensorimotor cortex [10].

The cause of DAN degeneration is yet unknown, but aging as well as a combination of genetic predisposition and environmental conditions are assumed to represent dominant risk factors for the development of PD [11]. A genome-wide association study by Satake and colleagues identified variances in the gene coding for human alpha-synuclein (hα-syn)—a mainly presynaptically localized protein—as one of the major genetic PD risk factors [12]. Mutations in this gene, as well as duplication or triplication of the coding sequence, have been assigned to the inherited forms of PD [13,14,15]. The hα-syn is one of the major components of Lewy bodies, a major cell-pathological hallmark of the disease. Increased hα-syn accumulation leads to DAN loss (summarized in [11]).

The role of DA in behavior modulation displays striking similarities between mammals and *Drosophila*. DA signaling in the rodent ventral striatum was associated with an important role in associative reward learning [16,17,18,19,20] and DA deficient mice display defective fear conditioning indicating the importance of DA in aversive learning [21]. Accordingly, it was found that DA deficient *Drosophila* cannot perform aversive classical conditioning [22] and since Schwärzel and colleagues [23] for the first time demonstrated the importance of DA for associative learning, many studies demonstrated and deciphered the DA circuits underlying learning in *Drosophila* (summarized in [24]). Another striking similarity between *Drosophila* and vertebrates is the impact of DA on feeding behavior. Like observations in mice where dopamine deficiency provokes hippophagy [25], DA deficient *Drosophila* display strongly reduced feeding behavior [22]. Further, DA plays an important role in the modulation of sleeping behavior in insects (summarized in [26]) and DA transporter mutant flies display a strong reduction in sleep [27] that corresponds to the increased waking bouts in DA transporter mutant mice [28]. The strong impact of DA in the regulation of sleeping behavior may partly explain why most PD patients suffer from sleep disorders [29,30,31].

DA signaling plays a decisive role in locomotion control in vertebrates as well as in invertebrates [22,32,33,34,35]. Since Feany and Bender established the first transgenic animal model of PD through ectopic hα-syn expression in *Drosophila* DANs [36], the fruit fly has been proven to be a valid model for investigating cellular as well as mechanistic processes in the development of the disease and attracted diverse studies [36,37,38,39,40,41,42,43,44,45,46,47,48,49]. However, unlike in human patients, no unilateral symptoms have been reported in the *Drosophila* PD disease model.

To investigate whether *Drosophila* DANs may display unilateral predominance in PD, we focused on a specific type of DANs, the PPL204 neurons, that symmetrically innervate the Antler (ATL) neuropil of both sides of the fly brain. Because ATL is morphologically isolated from the rest of the fly brain, precise analysis of asymmetry was possible. By visualizing the putative presynaptic sites of PPL204 DANs in the presence of the mutant form of hα-syn, we found a strongly unilateral predominance of presynaptic integrity impairment, a unilateral symptom that was for the first time described in *Drosophila* PD models.

## 2. Results

### 2.1. Characteristic Subsets of DANs Provide Symmetric Innervations to the Antler

**Figure 1:**
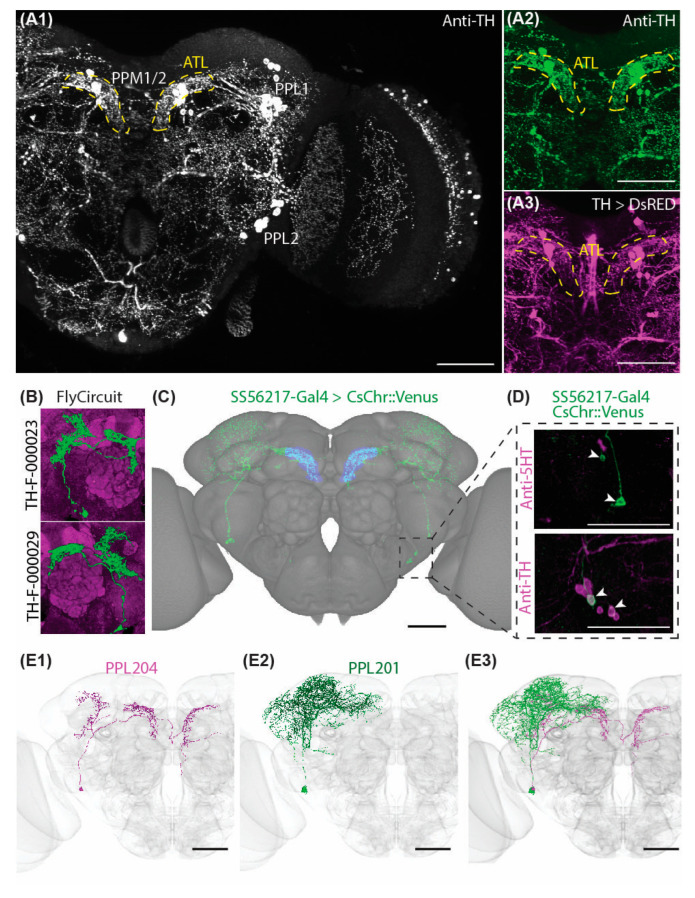
Symmetric Innervation Pattern of dopamine neurons (DANs) in the Antler (ATL) neuropil. (**A1**) In situ immunolabeling of the posterior half of a whole-mount brain with anti-tyrosine hydroxylase (TH) antibody, showing the protocerebral posterior medial 1/2 (PPM1/2) as well as the protocerebral posterior lateral 1 and 2 (PPL1, PPL2) clusters of DANs. Yellow dashed lines denote the location of ATL. (**A2**,**A3**) Co-localization of anti-TH immunolabeling and TH-Gal4-driven genetic labeling of DANs in the ATL (yellow dashed line). (**B**) FlyCiruit candidate DANs innervating the ATL. (**C**) Projection pattern of the neurons labeled in the SS56217 Split-Gal4 driver line, visualized with CsChrimson::mVenus and registered to the JRC2018 unisex brain template. Two cells are labeled. Blue indicates arborization in the ATL, whereas green indicates arborization elsewhere in the brain. Only one of the two cells innervate the ATL. (**D**) Co-localization of SS56217 Split-Gal4 (green) and anti-5-HT (top) or anti-TH (bottom) immunolabeling (magenta). SS56217 Split-Gal4 driven CsChrimson::mVenus expressing somata (arrowheads) do not overlap with anti-5HT immune-reactive cells but exclusively with two anti-TH immune-reactive cells, indicating that the targeted cells are DANs. (**E**) The expression pattern of SS56217 Split-Gal4 driver line overlaps with two PPL2 candidate DANs (PPL201—(**E1**), magenta and PPL204—(**E2**), green; (**E3**)—merge) identified data from FlyEM hemibrain, superimposed onto the common JRC2018 unisex brain template. Scale bars: 50 µm.

To identify a model system of *Drosophila* DAN for investigating potential unilateral predominance during the early stages of PD-associated neurodegeneration, we first screened for DANs that innervate both brain sides with a strong level of arborization symmetry in their target neuropils. Analysis of the antibody labeling against tyrosine hydroxylase (TH), the rate-limiting enzyme of DA biosynthesis, as well as the expression driver line under control of TH regulatory region (TH-Gal4), revealed that the ATL (brain structure in the posterior dorsomedial protocerebrum) is symmetrically innervated by characteristic subsets of DANs (Figure 1A). To identify the specific DANs that innervate this brain neuropil, we screened the FlyCircuit database [50] and found that the FlyCircuit neurons TH-F-000023 and TH-F-000029 are likely to contribute to the observed ATL arborization (Figure 1B). These are a pair of neurons, one neuron on each side, and their somata belong to the protocerebral posterior lateral type 2 (PPL2) cluster.

The neurons that morphologically match these cells have been identified as PPL204 in the recently published electron microscopy (EM) connectome database FlyEM Hemibrain [51]. To drive gene expression specifically in the PPL204 neurons, we generated and screened Split-Gal4 driver lines and obtained the line SS56217, which is a combination of the GAL4 activation domain (AD) in the RTH (VK000027) and GAL4 DNA binding domain (DBD) in the R42H01 (attP2) (Figure 1C). This AD / DBD combination resulted in a very specific expression in two neurons per side of the PPL2 cluster. One of the two neurons innervates ATL, and the other sends its projections solely outside the ATL, mainly to the superior lateral protocerebrum (SLP), superior intermediate protocerebrum (SIP), superior medial protocerebrum (SMP), lateral horn (LH), superior clamp (SCL), crepine (CRE), and mushroom body (MB) calyx. Double labeling of SS56217-Gal4-driven expression and immunolabeling of anti-TH overlapped for both cells, demonstrating that both neuron types labeled by SS56217-Gal4 are DANs (Figure 1D). Morphological matching of the two PPL2 DANs targeted by SS56217 Split-Gal4 driver line against candidate DANs identified data from FlyEM hemibrain [51] revealed that the ATL-innervating neuron corresponds to PPL204, whereas the other corresponds to PPL201 [51] (Figure 1E1–E3). The two PPL2 DANs do not display any synaptic interaction among each other except for one synaptic connection outside the ATL (Supplementary Figure S1A,B).

### 2.2. Hα-SynA30P Expression Induces Age-Dependent Presynapse Depletion of ATL DANs

Ectopic expression of a mutant form of human α-synuclein hα-synA30P in *Drosophila* induces alterations in the cytoskeleton and mitochondria already in pre-symptomatic *Drosophila* and change substantially across the lifespan [45,46,47]. However, Chen and colleagues showed that soluble hα-syn oligomers phosphorylated at Tyr125 were more abundant in young flies and that blocking phosphorylation of Tyr125 increases hα-syn toxicity [52]. In contrast, it appears that phosphorylation of hα-syn at Ser129 increases the formation of toxic oligomers [52].

DANs appear very vulnerable to ectopic human α-synuclein hα-synA30P expression and were reported to suffer from neurodegeneration [21] and a significant decrease in synaptic connections to their target neuropils [28] This apparent reduction in neuronal functionality is accompanied by strong locomotor deficits in climbing behavior during aging [36,43].

Using the GRASP-technique to visualize the interaction between DANs and MB neurons [53], we previously observed that hα-synA30P expression in combination with aging negatively affects PAM-cluster DAN connection to their target region in the MB medial lobes [43]. However, potentially due to the massive vulnerability of PAM DANs or to the GRASP technique that was employed to visualize PAM-MB interaction in this study, we did not observe distinct asymmetric phenotypes [28].

Studies on human patients and vertebrate models for PD showed that DAN dysfunction displays a strong lateral predominance [7,10].

To investigate whether hα-synA30P expression in *Drosophila* may indeed affect DANs with unilateral predominance, we expressed an HA-tagged version of a synaptic vesicle-associated protein synaptotagmin (syt::HA) together with hα-synA30P and monitored the distribution of presynaptic signals in PPL204 DANs in the symmetrically innervated ATL of aging flies. To distinguish between normal and pathological aging, we first quantified changes in presynaptic syt::HA expression in young (5-day-old) flies to the expression in aged (30-day-old) flies.

**Figure 2:**
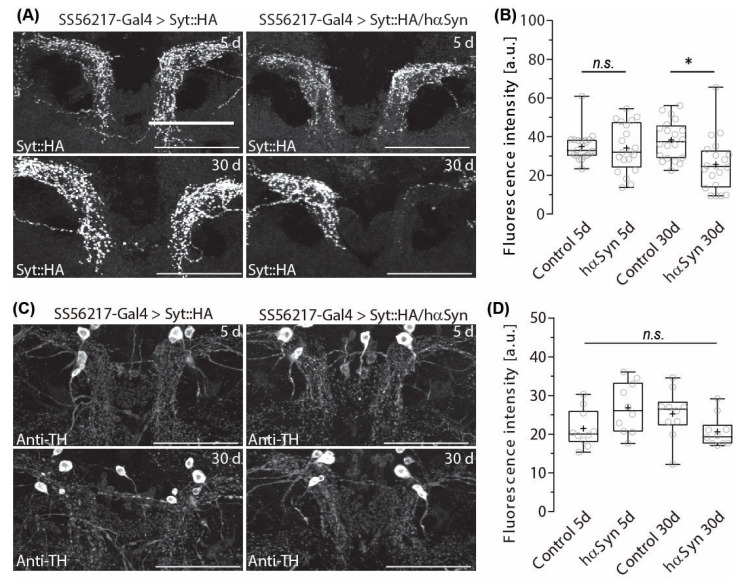
Age-dependent presynapse depletion of PPL204 DANs induced by Hα-SynA30P expression. (**A**) Signal intensity of presynaptic syt::HA immunoreactivity in 5- and 30-day-old control flies and the flies that ectopically express hα-SynA30P in PPL204 DANs. A representative image is presented for each case. Note the drastically sparser signals on the left side of brain in bottom right image. (**B**) Fluorescence intensity, measured as the average signal levels of the voxels inside the volume of ATL. a.u., artificial unit. Open circles denote values from each brain side (*n* = 20) of individual samples (*N* = 10). Box plots denote the 0, 25, 50, 75 and 100 percentiles. + indicates the mean value. * *p* < 0.05; n.s. *p* > 0.05; Shapiro–Wilk normality Test followed by a Kruskal–Wallis ANOVA with Dunn’s correction. (**C**) TH immunolabeling in the ATL in 5- and 30-day-old control flies and the flies that ectopically express hαSynA30P in PPL204 DANs. Same samples are shown for each case as in (**A**). Note that the signals in the bottom right panel are not reduced on both sides, in contrast to the corresponding image in (**A**). (**D**) Fluorescence intensity measured and averaged in the same way as in (**B**). Open circles denote values from each brain side (*n* = 10) of individual samples (*N* = 5). Scale bar: 50 µm for all panels.

Fluorescence analysis of syt::HA antibody labeling revealed that PPL204 neurons display a significant age-dependent reduction in the signal distribution in the ATL of the flies expressing hα-synA30P when compared to control flies of the same age (Figure 2A). Quantitative signal intensity comparison revealed that, whereas 5-day-old flies expressing hα-synA30P displayed comparable syt::HA signal intensity to control animals without hα-synA30P expression, 30-day-old flies expressing hα-synA30P showed a significant reduction in presynaptic signal intensity, indicating synaptic depletion (Figure 2B).

In human patients, hα-synA30P oligomers, as well as aggregating protofibrils, can accumulate in synapses, which disrupt the maintenance of synapse functionality resulting in a synaptopathic dying back of DANs from impaired synapses to somata (reviewed in [54]). Ectopic expression of hα-synA30P was reported to result in loss of DANs in 30-day-old flies [36]. On this account, we addressed the question of whether the observed reduction of putative presynaptic structures should be the result of the degeneration of entire neurons or a kind of synaptopathic phenotype. To examine whether hα-synA30P expression affects the overall neuronal structure or only putative presynapse distribution, we labeled DANs with an antibody against TH.

The ATL neuropil is innervated by PPL204 DAN and apparently another DAN with similar arborization densities. If PPL204 neuronal fibers degenerate because of ectopic hα-synA30P expression, ATL DAN arborizations in the ATL should become significantly sparser.

However, TH antibody labeling revealed that the overall DAN arborization patterns in the ATL of 30-day-old flies were not evidently affected by hα-synA30P expression in PPL204 (Figure 2C) and quantitative comparison of signal intensity did not show any significant decrease (Figure 2D). These findings strongly suggest that TH immunoreactivity in the ATL may be compensated by ATL innervating DANs other than PPL204. We screened for additional potential candidates for DANs that send their projections to the ATL and compared potential candidates in the EM data set to immunohistochemical labeling against TH and identified ATL042 as a potential candidate for an additional ATL DAN of the PPL2 cluster (Supplementary Figure S2A–C).

The expression of hα-synA30P caused not only synapse depletion in aged flies; the phenotype shows striking left-right asymmetry (Bottom-right panel of Figure 2A). Among the ten 30-day-old brain samples we examined, seven samples showed a much more significant loss of presynaptic signals on the left side of the brain (right side of the image), whereas one sample showed a stronger reduction on the right-brain side. Only two samples showed a roughly equal density of presynaptic signals on both sides; their densities were either unchanged compared to young flies, suggesting that they were not affected sufficiently by hα-synA30P expression, or strongly reduced on both sides, suggesting a symmetric effect of hα-synA30P expression.

**Figure 3:**
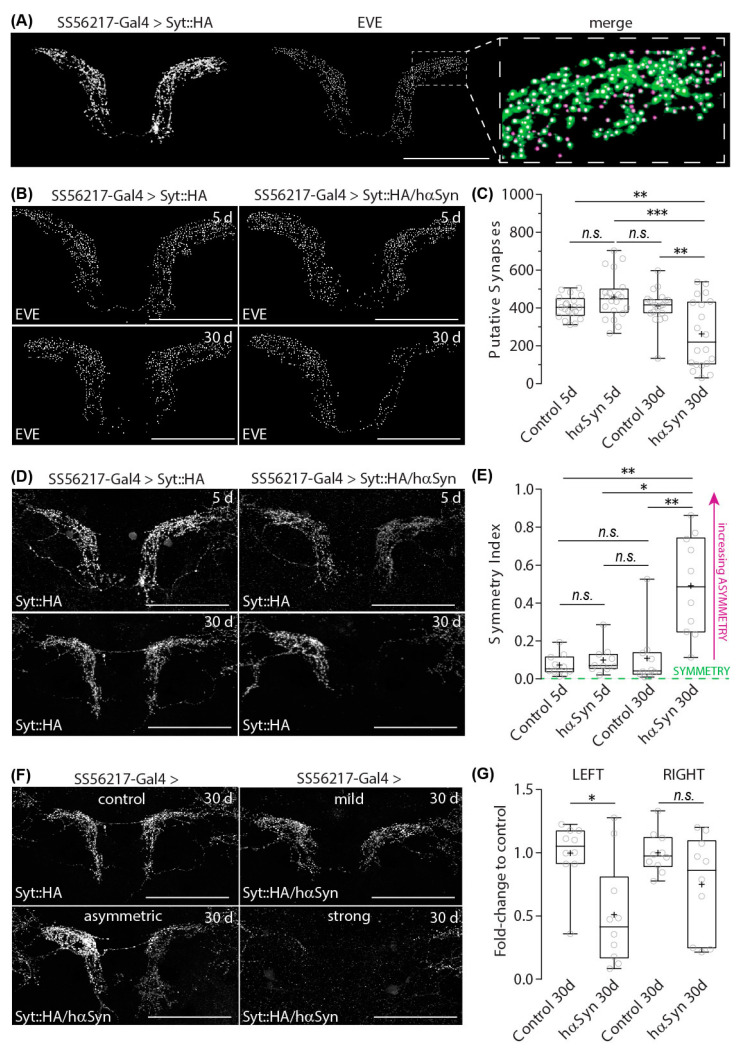
Presynapses numbers of PPL204 DANs in the ATL. (**A**) Labeling of putative presynapses of PPL204 DANs in ATL, visualized with presynapse-targeted syt::HA reporter expression under control of SS56217-Gal4 (right). Automatized detection of labeled objects using Excluded Volume Embedding (EVE) algorithm (middle). Overlay of syt::HA labeling (green) and EVE-detected objects (magenta); blow up view of a small portion of ATL (right). Note that individual green signals of boutons are marked with magenta dots of EVE detection. (**B**) EVE-detected syt::HA labels in 5- and 30-day-old control flies and flies that ectopically express hαSynA30P in PPL204 DANs. (**C**) Counts of EVE-detected labels. Open circles denote values from each brain side (*n* = 20) of individual samples (*N* = 10). Box plots denote the 0, 25, 50, 75 and 100 percentiles. + indicate the mean value. *** *p* < 0.001; ** *p* < 0.01; * *p* < 0.05; n.s. *p* > 0.05; Shapiro–Wilk normality Test followed by a Kruskal–Wallis ANOVA with Dunn’s correction. (**D**) Presynaptic syt::HA immunoreactivity in 5- and 30-day-old control flies and flies that ectopically express hαSynA30P in PPL204 DANs. (**E**) Asymmetry Index for evaluating the degree of left-right asymmetry of presynapse counts, calculated as [Synapses (Left)—Synapses (right)]/Synapses (total). Open circles denote values from individual samples (*N* = 10). (**F**) Various penetration degrees of hαSynA30P-induced age-dependent depletion. Presynaptic labeling with syt::HA. Representative images for four levels of depletion are presented. (**G**) Ratio between the EVE-quantified presynapse counts of 30-day-old control flies and 30-day-old flies expressing hαSynA30P in PPL204 DANs on the same brain side. Open circles denote values from individual samples (*N* = 10). Scale bar: 50 µm in (**A**) for all panels.

To characterize this putative asymmetry, we employed a more precise quantitative approach to count the actual number of presynaptic densities using an automated object-counting algorithm of Excluded Volume Embedding (EVE) [55]. A comparison of the original image and the spherical objects detected by EVE showed a very high correlation (Figure 3A).

In agreement with our fluorescence intensity analysis (Figure 2A,B), counted numbers of presynaptic structures of PPL204 DAN showed a significant reduction in the aged flies with hα-synA30P (Figure 3B,C). To quantify the lateral asymmetry of syt::HA labeled putative presynaptic structures between brain sides (Figure 3D), we calculated an asymmetry index as the ratio of the absolute difference of EVE-quantified presynapse counts between brain sides in relation to the total counts on both sides (Figure 3E). Five- and 30-day-old control flies as well as 5-day-old flies expressing hα-synA30P display a very low asymmetry index, reflecting a strongly reliable symmetry of putative presynapses between brain sides. In contrast, the significantly increased asymmetry index of 30-day-old hα-synA30P-expressing flies indicates a strong lateral predominance in presynapse depletion on one brain side.

This laterality of putative presynapse density does not appear to represent an innate asymmetric innervation of PPL204 DANs to the ATL over the population. Syt::HA-labeled putative presynapses in the ATL in 5-day-old flies appear equilibrated between both brain sides (Figure 3D, top right). The observed difference in synapse numbers between the right- and left-brain sides only developed during aging in the presence of hα-synA30P (Figure 3D, bottom right). This represents a significant lateral predominance in presynapse depletion and might be comparable to the laterality of DAN degeneration described for human patients. However, it is worth noting that, e.g., the communication between the insula and sensorimotor cortex is altered in PD patients provoking hemispheric asymmetry that relates to non-motor symptoms [10]. We, therefore, asked whether asymmetric presynapse depletion exclusively in ATL PPL204 neurons affects spontaneous locomotor behavior (Supplementary Figure S3A). Our detailed analysis indicates that neither young nor aged flies expressing hα-synA30P in PPL204 show significant alteration, either in the traveled distance (Supplementary Figure S3B) and velocity (Supplementary Figure S3C) and active velocity (Supplementary Figure S3D), or in the activity index (Supplementary Figure S3E), indicating that the presence of hα-synA30P in these neurons does not induce alterations of spontaneous horizontal locomotor (Supplementary Figure S3A–D).

It is worth noting that the penetrance of the impact of hα-synA30P expression in synapse depletion varies. The impact of hα-synA30P in PPL204 DANs in aged flies can reach from very mild penetrance with an appearance that is comparable to control flies to intermediate penetrance with a strong asymmetric appearance of presynaptic labeling to severe penetrance where presynapses of PPL204 DANs in ATL appear nearly completely depleted on one side (Figure 3F). As described earlier, the presynapses on the left-brain side appeared to be affected with higher penetrance than the presynapses on the right brain side. To quantify this effect, we calculated the ratio between the number of presynaptic syt::HA labeling in the PPL204 DANs of individual 30-day-old flies expressing hαSynA30P in PPL204 DANs to the mean number of labeling in the 30-day-old flies under the corresponding experimental conditions for each brain side. This revealed that the PPL204 presynapses of hα-synA30P-expressing flies are significantly reduced on the left but not on the right side (Figure 3G). Importantly, ATL is contributed by a pair of PPL204 neurons arising from each side of the brain. Thus, synaptic depletion occurs on the same brain side regardless of whether it is ipsilateral or contralateral to the soma location.

The SS56217-Gal4 driver line labels not only PPL204 DAN but also PPL201 DAN, which arborizes more broadly in various parts of the brain. Because of the weaker labeling intensity and sparser arborization patterns, we were not able to obtain reliable data about its potential presynapse depletion and laterality.

### 2.3. Connectome Characterization of Implicated PPL204 Neuronal Circuits

PPL204 DANs exhibit clear lateral predominance in the induced PD model. The function of these neurons, however, is hardly known so far. We, therefore, performed several analyses to address this problem.

**Figure 4:**
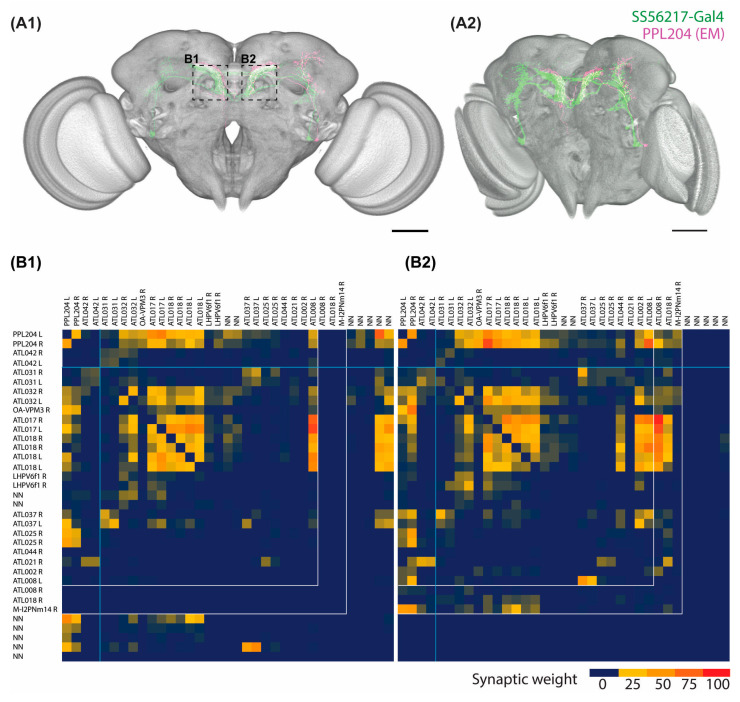
Synaptic connectivity of PPL204 neurons. (**A**) Posterior (**A1**) and posterior-lateral (**A2**) views slightly dorsally tilted of the light-microscopy data of the neurons labeled with SS56217-Gal4 and UAS-CsChrimson::mVenus (green) and segmented electron-microscopy data of the PPL204 neuron (magenta, data from FlyEM hemibrain), superimposed onto the common JRC2018 unisex brain template. (**B**) Connectivity matrix of the PPL204 DANs and strongly interconnected neurons within the left ATL (**B1**) and right ATL (**B2**). Top row and leftmost column list the neuron names that appear in the FlyEM hemibrain version 1.2 database. “NN”, not named yet. “R” and “L” denote the neurons arising from the right- and left-brain sides, respectively. The color code of each cell indicates the number of detected connections from the neuron listed on the left column to the neuron listed on the top row. Thus, for the two PPL204 neurons, the top two rows of the matrix indicate their putative downstream partners, whereas the left columns indicate their putative upstream partners. Scale bar: 50 µm.

As explained before, the characteristic projection pattern of these neurons has been identified as PPL204 in the FlyEM hemibrain database [51] (Figure 4A). To understand how alterations in PPL204 DAN signaling may be influenced by the lateral predominance of presynapse depletion, we analyzed the neuronal connectivity of these neurons in the ATL using NeuPrint [56]. For our analysis we included the identified candidates for DANs and other neurons with connections of at least 10 synapses, as connections with 3–9 synapses were previously reported as medium strength connections and connections with 10 or more synapses were reported as strong, reflecting a high level of reliability [51].

We generated connectivity heat map matrices of the two PPL204 DANs arising from both sides (the first two rows and columns of the matrices) and the neurons that have strong connections with PPL204 in the ATL of each brain side (Figure 4B1,B2). PPL204 DANs give output signals to a tyraminergic/octopaminergic modulatory neuron OA-VPM3, various neurons that primarily arborize in the ATL (ATL-named neurons), a few neurons that arborize mainly in the LH (LHPV6f1) and the lateral accessory lobe (LAL148), one antennal lobe projection neuron (M-l2PNm14), and a few neurons that have not been named (NN) because of the lack of soma location information inside the hemibrain EM volume (top two rows of the heat map). These downstream partners are the primary candidates that might be affected by the hα-synA30P-induced presynaptic depletion. PPL204 DANs receive reciprocal inputs from OA-VPM3 and several ATL neurons as well as unilateral inputs from several other ATL neurons and unnamed neurons (left two columns of the heat map). Our connectome analysis of PPL204 further indicates that two yet unidentified neurons with strong pre-and postsynaptic connections to PPL204, namely ATL002 and ATL008, show strong asymmetry in their synaptic contacts with PPL204 between left and right ATL (Supplementary Figure S4). In this regard, it is interesting that ATL002 displays much stronger laterality in both pre- and postsynaptic connection to the left ATL, whereas ATL008, in contrast, displays stronger in- and output to the right ATL (Supplementary Figure S4).

### 2.4. PPL204 Presynapse Depletion Does Not Affect 5-HT Immune-Reactivity in the ATL

Previously, we showed that 5-HT Neurons (5-HTNs) densely innervate ATL [57]. Alterations in DA signaling have been shown to provoke opponent effects on 5-HTNs in vertebrates [58] and invertebrates [57]. The latter study analyzed the flies that cannot produce DA throughout the brain. In contrast, here we are interested in how gradually progressing DAN presynapse depletion (Figure 2A,B) in yet structurally unaffected DAN fibers (Figure 2C,D) may alter 5-HT signaling.

**Figure 5:**
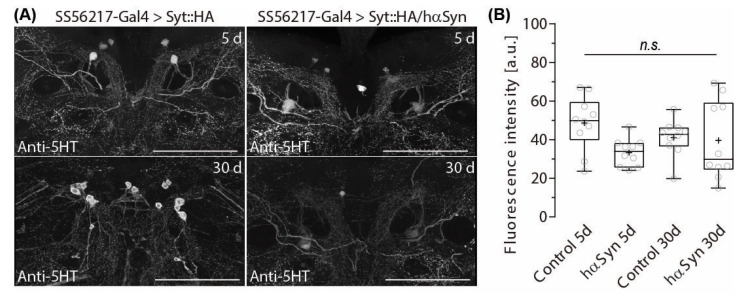
PPL204 neuron presynapse depletion does not affect 5-HT immunoreactivity in the ATL. (**A**) 5-HT immunolabeling in the ATL in 5- and 30-day-old control flies and flies that ectopically express hαSynA30P in PPL204 DANs. (**B**) Fluorescence intensity of anti-5-HT immunolabeling, measured as the average of signal levels of the voxels inside the volume of ATL. Open circles denote values from each brain side (*n* = 10) of individual samples (*N* = 5). Box plots denote the 0, 25, 50, 75 and 100 percentiles. + indicate the mean value. n.s., *p* > 0.05; Shapiro–Wilk normality Test followed by a Kruskal–Wallis ANOVA with Dunn’s correction. Scale bar: 50 µm.

To this end, we ectopically expressed hα-synA30P in PPL204 DANs and measured the fluorescence intensity of anti-5-HT immunolabelling in ATL in 5- and 30-day-old flies. We found that despite the strong 5-HT immunolabelling in the ATL (Figure 5A), hα-synA30P-triggered presynapse depletion in PPL204 DANs does not trigger alterations in anti-5-HT immunoreactivity in the ATL (Figure 5A,B). Current NeuPrint data do not include information about their matching neurons, but based on our findings we suspect that the depleted DA output from PPL204 DANs in the induced PD-model condition may not directly interact with ATL 5-HTNs or may be compensated by other DANs innervating the ATL such as ATL042.

As explained earlier, the presynapse density and counts of PPL204 neurons are essentially symmetric in young 5-day-old control flies without hα-synA30P expression (Figure 3E). Yet, the FlyEM hemibrain electron microscopy data obtained from a fly under a comparable condition display a slight asymmetric distribution; about 30% more synapses were detected in the ATL on the right-brain side than on the left (Table 1). The EVE-based presynapse counts (Table 1, for right and left PPL204s combined) are generally much fewer than the presynapse count in the hemibrain EM data. This is because syt::HA labels an entire synaptic bouton as a fused mass, whereas each bouton contains several presynaptic T-bars that are individually detected and counted in the EM data. The mean value of EVE count in the ATL of 10 5-day-old flies was essentially similar between brain sides, but this includes individual variability. Three out of 10 analyzed samples showed 20–30% more synapses on the right side, such as the fly analyzed in the hemibrain EM data. Thus, it is very likely that the observed asymmetry in the EM data are because of individual variability, although there is some possibility that the number of presynaptic T-bars may display asymmetry even when the number of presynaptic boutons is essentially symmetric.

In principle, stochastic mechanisms in neuronal circuit wiring and the resulting left-right asymmetry are determinants for interindividual behavioral variability [59]. Such neuronally implemented handedness may facilitate behavioral decisions in the absence of asymmetric stimuli or prevent potential unfavorable response delays under equivalent stimuli from the environment [60]. Thus, the laterality of synaptic connectivity weight in the ATL may be highly individual and may not be adequately represented in a single EM dataset.

## 3. Discussion

In this study, we found a single pair of neurons that develop a significant lateral predominance in presynapse depletion under the model PD condition. A PPL204 neuron forms symmetric arborization on both sides of the ATL neuropil. Ectopic hα-synA30P expression induces presynapse depletion with a significant lateral predominance on one brain side in aging flies. These appear to be comparable to the laterality of DAN degeneration described for human patients [5,7,10,61].

Most neuropils of the *Drosophila* brain appear continuous with no clear boundaries. The MB, the central complex, and the antennal lobe are major exceptions in that the structures are separated with glial sheaths from the surrounding neuropils. The ATL is another exception, it protrudes from the posterior surface of the fly brain neuropils and extends through the cell body rind, also called the cortex [62]. Whereas many DANs in the fly brain have broad arborization, the DANs in the ATL have tightly packed branches. These enabled a clear distinction and quantification of their morphological changes. Similar laterality analyses of other DANs are technically more complex, but not impossible. Our findings will raise the importance of such studies in the future.

Structural lateral predominance in the *Drosophila* brain and the role of such asymmetry in brain functionality have been described for a couple of neurons. For example, more than 90% of flies show a 10-µm diameter asymmetrical glomerulus-like structure on the right side of the central complex, known as the asymmetric body. Although the remaining 10% of flies display symmetry in this brain region, its asymmetry appears to be necessary for the formation and recall of long-term memories [63]. It has also been described that the antenna-mediated flight control in flies reveals a forceful asymmetry [64], which potentially facilitates stable odor tracking and may help increase the efficiency of search behavior in a multisensory environment [65]. Unlike these neurons, the syt::HA visualized boutons of PPL204 do not show any significant laterality in wild-type flies as well as at the beginning of the PD-model condition. The laterality is the outcome of the long-term existence of hα-synA30P proteins. The reason why such presynapse depletion exhibits lateral predominance remains unclear. The phenotype showed a broad spectrum, from nearly symmetric to strong laterality to almost complete loss on both sides; 8 out of 10 observed flies showed clear asymmetry, suggesting that it is dependent on yet unknown individual factors that favor the unilateral acceleration of presynapse depletion in many animals.

Our study indicates that lateral predominance in neurodegeneration is not restricted to vertebrates but occurs also in insects. The lateral predominance of DAN degeneration in humans is putatively related to patient handedness, as right-handed patients display an accelerated depletion of putamen DANs on the contralateral brain hemisphere [66]. In *Drosophila*, Versace and colleagues observed a very small but significant overall population preference for anticlockwise circling [67]. This may indicate a general bias to right-handed movement initiation in *Drosophila*. However, whether and how lateral predominance in the handedness of behavioral output control may be related to the laterality in degeneration, and the neuronal mechanisms underlying the asymmetric synapse degeneration, need further investigation.

The unimagined similarity in the asymmetry of disease-triggered neuronal dysfunction between flies and vertebrate models paves the way to a better understanding of the mechanisms underlying lateral predominance in the progression of degenerative phenotypes in Parkinson’s disease as well as in other neurological disorders that also exhibit lateral predominance.

## 4. Materials and Methods

### 4.1. Drosophila Strains and Maintenance

If not stated elsewhere, fly stocks were raised and crossed at 25 °C and 50% humidity on standard corn meal/yeast/agar under a 12 h/12 h light/dark cycle. The following fly strains were used: w; UAS-hαSynA30P [36], 20xUAS-CsChrimson-mVenus (shortened as CsChr::Venus in figures) in attp18 [68], UAS-DsRed (C6) [69] elav-GAL4 (elav^C155^, Bloomington Stock center, #458), wild-type Canton S (the line inherited from Seymour Benzer lab via Yoshiki Hotta lab), yw; UAS-Syt::HA; sp/CyO. The combination of the strains UAS-Syt::HA with UAS-hαSynA30P (shortened as SytHA/hαSyn in figures) was obtained and stabilized through double balancers of the second and third chromosomes. The stabilized Split-GAL4 driver line SS56217-Gal4 was obtained through the combination of RTH-GAL4 AD in VK000027 with R42H01-Gal4 DBD in attp2.

### 4.2. Sample Preparation

Brain tissues were dissected on ice in 1× PBS, fixed in 2% PFA for 1 h at room temperature and washed four times 15 min at room temperature with PBS containing 0.6% Triton-X (PBT). After blocking the tissues were incubated in 5% normal goat serum (NGS) overnight at 4 °C, incubated in 5% NGS containing primary antibodies (mouse anti-Brp [70] 1:5, Developmental Studies Hybridoma Bank (DSHB); rabbit anti-TH, 1:50, Merck, Rahway, NJ, USA; rat anti-5-HT, 1:50, abcam, Cambridge, UK; rabbit anti-HA, 1:300, Cell signaling Technology, Danvers, MA, USA; rat anti-HA, 1:300, Sigma-Aldrich, St. Louis, MO, USA; chicken anti-DsRed, 1:200, Rockland) for 4 h at room temperature and two overnights at 4 °C, and subsequently washed four times 15 min in PBT at room temperature. Specimens were then incubated in PBT containing 5% NGS and corresponding secondary antibodies (goat anti-mouse Alexa Fluor 647, 1:500, Thermo Fisher Scientific, Waltham, MA, USA; goat anti-rabbit AF488, 1:500, Cell Signaling Technology; goat anti-rat AF488, 1:500, Thermo Fisher Scientific; goat anti-mouse AF568, 1:500, Thermo Fisher Scientific; goat anti-rabbit AF647, 1:500, Thermo Fisher Scientific; goat anti-rat AF647, 1:500, Thermo Fisher Scientific; goat anti-chicken AF568, 1:500, Thermo Fisher Scientific) for two to three overnights at 4 °C and washed four times 15 min at room temperature in PBT. The tissues were then fixed for the second time in 2% PFA for 4 h at room temperature and washed four times 15 min in PBT and once with 1 mL PBS for 10 min at room temperature. After the final wash, specimens were mounted on poly-L-lysine (PLL)-coated cover glasses, and dehydrated through a series of 10 min incubations in EtOH with increasing concentrations (30%, 50%, 75%, 95%, and three times 100%), incubated three times 5 min in xylene for clearing, and finally embedded in DPX, as described in the FlyLight Protocol.

### 4.3. Image Acquisition and Processing

After DPX polymerization, specimens were imaged using an Olympus Fluoview FV1000 Confocal Laser Scanning Microscope with an Olympus UPlanFLN 40×/1.3 Oil objective at a z-step interval of 0.5 µm and a resolution of 1600 × 1600. To obtain the entire image of the brain, the samples were scanned in three tiles.

After acquisition tiles were stitched with the Fiji [71] (Version 1.52p) “Pairwise Stitching” plugin [72] and pixel width, pixel height and voxel depth of the stitched data were adjusted to the image properties of original scans. Background signals were subtracted with the “Subtract Background” function of FIJI (rolling ball radius of 127 pixels), and then the “Enhance Local Contrast (CLAHE, Contrast Limited Adaptive Histogram Equalization)” plugin was run on the stack of images (block size = 127, histogram bins = 256, maximum slope = 2) on all channels. To allow for precise registration on the template brain, signals of the structures that are not in the template, e.g., ocelli, were manually removed using VVDViewer software [73] (https://github.com/takashi310/VVD_Viewer, released on 19 September 2022 (version 1.5.3), latest relevant updated for data analysis presented in this work on 9 December 2022 (version 1.5.7), last accessed on 5 May 2023, (version 1.6.4)). Subsequently, brains were registered to the JRC2018 unisex 63× brain template [74] using the “CMTK (Computational Morphometry Toolkit) registration runner” macro for FIJI (written by Sándor Kovács, using CMTK by Thorsten Rohlfing and Munger by Gregory Jefferis) running on an IBM x3850 x5 two-node 80-core server.

Three-dimensional morphologies of the registered neurons were visualized with VVDViewer; the settings for optimal visibility were adjusted, scalebars were added and images were saved using the “Capture” function.

Acquired images of brains of SS56217>DsRed flies were registered to Janelia Research Campus Unisex *Drosophila* Template brain (JRC2018) [74] using the anti-Brp channel as a reference.

### 4.4. Data Analysis

Registered 3D image stacks were converted to Color Depth Max Intensity Projection (ColorDepth MIP) images. Fiji plugin ColorMIP Mask Search [75] was used to screen against single neuron images of TH-Gal4>MARCM from FlyCircuit [50] database. This search is possible as original images of FlyCircuit were registered to their own template brain (FCWB) and transformed into the JRC2018 template brain [76]. Three-dimensional image stacks of found candidates were further compared to immunohistochemical labeling against TH in 3D render software VVDViewer (according to Section 4.3) [73].

For the Split-GAL4 line SS56217 flies expressing HA-tagged synaptotagmin and αSynA30P, presynapses in the ATL were detected and automatically counted using the Excluded Volume Embedding (EVE) algorithm [55] implemented in VVD Viewer.

For average HA-tag signal intensity measurements, Antler neuropils of each sample were manually segmented in VVDViewer based on anti-Brp labeling and average signal intensities were measured in ImageJ through the Stack.getStatistic function.

## Figures and Tables

**Table 1:** Presynapse numbers of PPL204 DANs in the ATL. Light microscopy data represent EVE-based counts of 10 brain samples. Electron microscopy data represent the counts of individual neurons in a single sample (Data from FlyEM hemibrain [51]).

	Light Microscopy (Bouton)	Electron Microscopy (T-Bar)		
	Mean ± SD of 10 Samples	PPL204_R	PPL204_L	Total
ATL (left)	435.2 ± 57.7	201	379	580
ATL (right)	374.6 ± 41.4	462	295	757

## Data Availability

Publicly available datasets analyzed in this study can be found here: FlyEM hemibrain (https://neuprint.janelia.org, last accessed on 5 May 2023), FlyCircuit (http://www.flycircuit.tw/, last accessed on 5 May 2023). Other data presented in this study are available on request from the corresponding author.

## Supplementary Materials

The following supporting information can be downloaded at: https://www.mdpi.com/article/10.3390/ijms24108585/s1. References [77,78,79] are cited in the supplementary materials.
