# Supplementary material for: Asymmetric Presynaptic Depletion of Dopamine Neurons in a Drosophila Model of Parkinson’s Disease
Source: Int J Mol Sci. 2023 May 11;24(10):8585. doi: 10.3390/ijms24108585 (PMC10218197; doi:10.3390/ijms24108585)
Supplement: Supplementary file 1 [file ijms-24-08585-s001.zip › ijms-2305857-supplementary.pdf]

# Asymmetric Presynaptic Depletion of Dopamine Neurons in a *Drosophila* Model of Parkinson's Disease

**Jiajun Zhang [1], Lucie Lentz [1], Jens Goldammer [1], Jessica Iliescu [1], Jun Tanimura [2,†] and Thomas Dieter Riemensperger [1,*]**

[1] Institute of Zoology, Experimental Morphology and Neuroanatomy, University of Cologne, Zuelpicher Str. 47b, 50674 Cologne, Germany

[2] Neuronal Circuit Division, Institute of Molecular and Cellular Biosciences, The University of Tokyo, 1-1-1 Yayoi, Bunkyo-ku, Tokyo 113-0032, Japan

* Correspondence: triemens@uni-koeln.de

† Current affiliation: Department of Neurology, Aizawa Hospital, 2-5-1 Honjo, Matsumoto 390-8510, Japan.

## Supplementary Materials

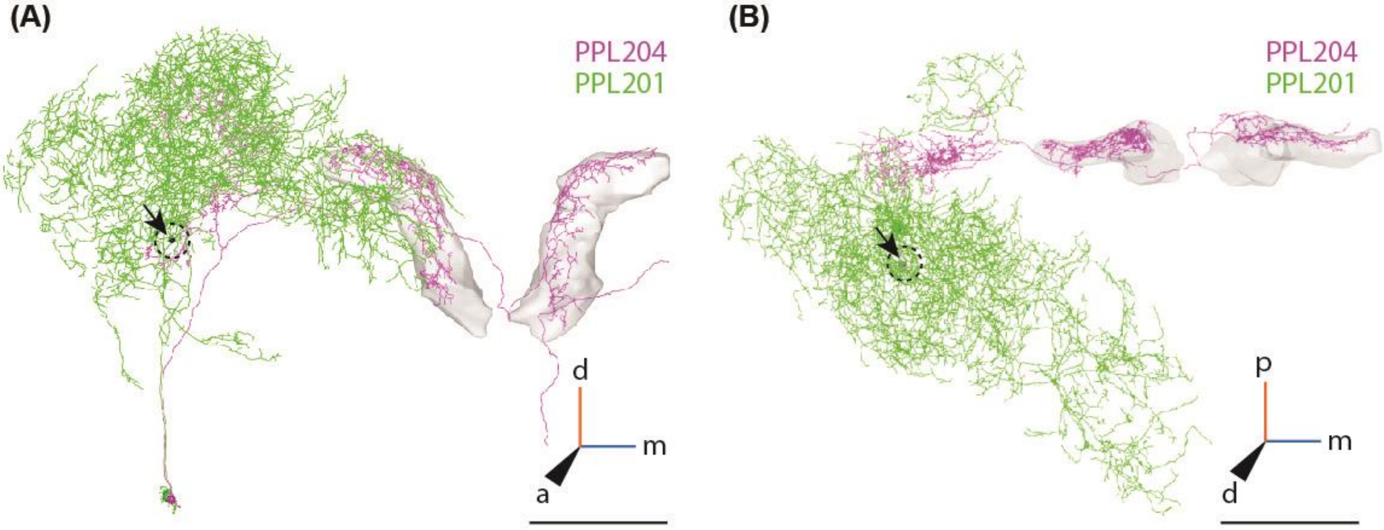


**Supplementary Figure S1: Synaptic connectivity between PPL201 and PPL204 neurons. A)** Frontal view on the two PPL2 candidate DANs, PPL201, green and PPL204 (identified data from FlyEM hemibrain): PPL201 (green) and PPL204 (magenta) show only one single synaptic connection (marked by the arrow in the circle) outside the ATL. **B)** Dorsal view on PPL201 and PPL204. Synaptic connection is marked by the arrow in the circle. Scale bars: 50 µm

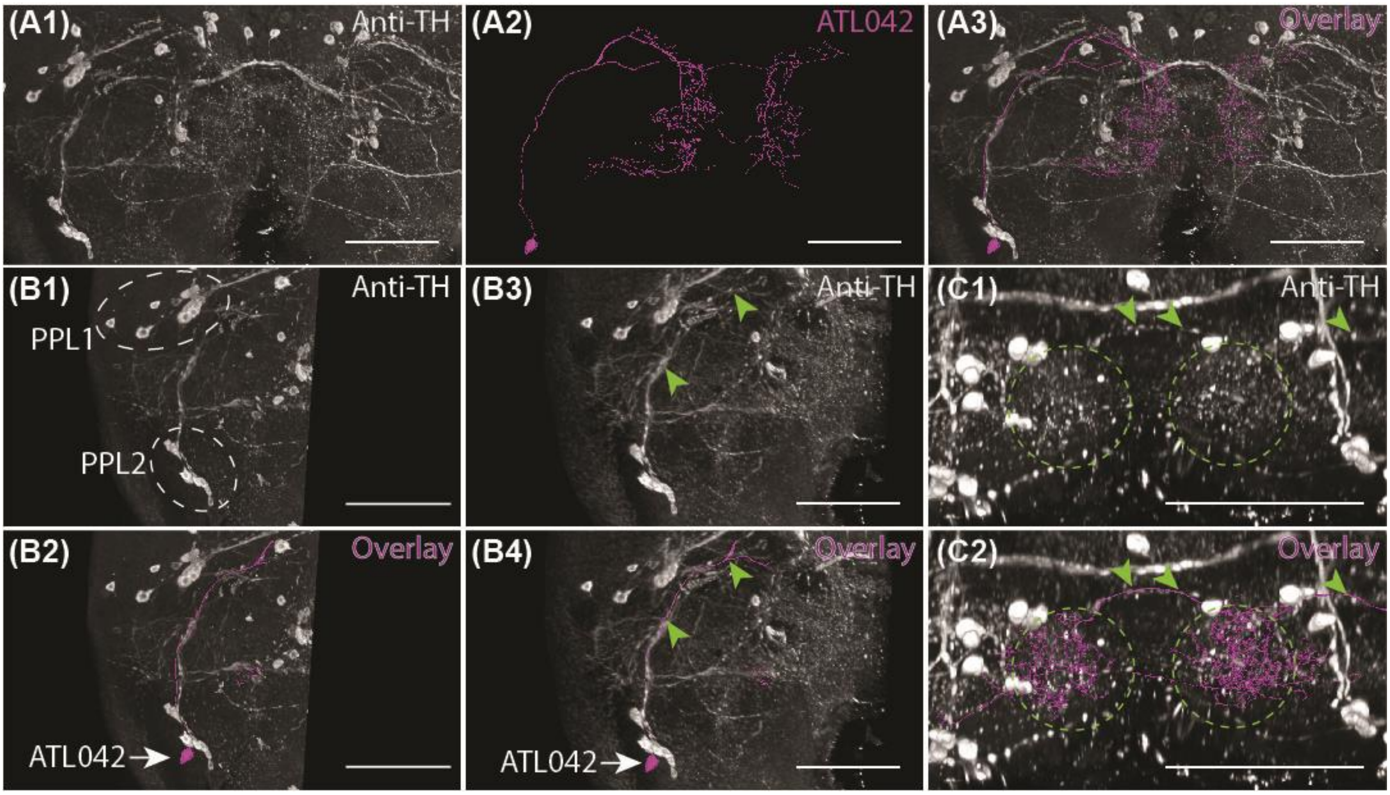


**Supplementary Figure S2: ATL042 is a candidate for PPL2 DANs. A)** Overlay of Anti-TH immunolabelling (A1) and the ATL042 EM data (A2) superimposed on the onto the common JRC2018 unisex brain template (A3) indicates strong overlap of TH immunoreactive cell body fibers. **B)** View on the Anti-TH immunoreactive cell body fiber of the PPL2 cluster (B1, B3, green arrow heads) and the merge between TH immunoreactive PPL2 cell body and ATL008 (B2, B4). **C)** View on the Anti-TH immunoreactive projections (green circles) and projections (green arrow heads, C1, C2) and the merge between TH immunoreactive PPL2 cell body and ATL008 (C1, C2).

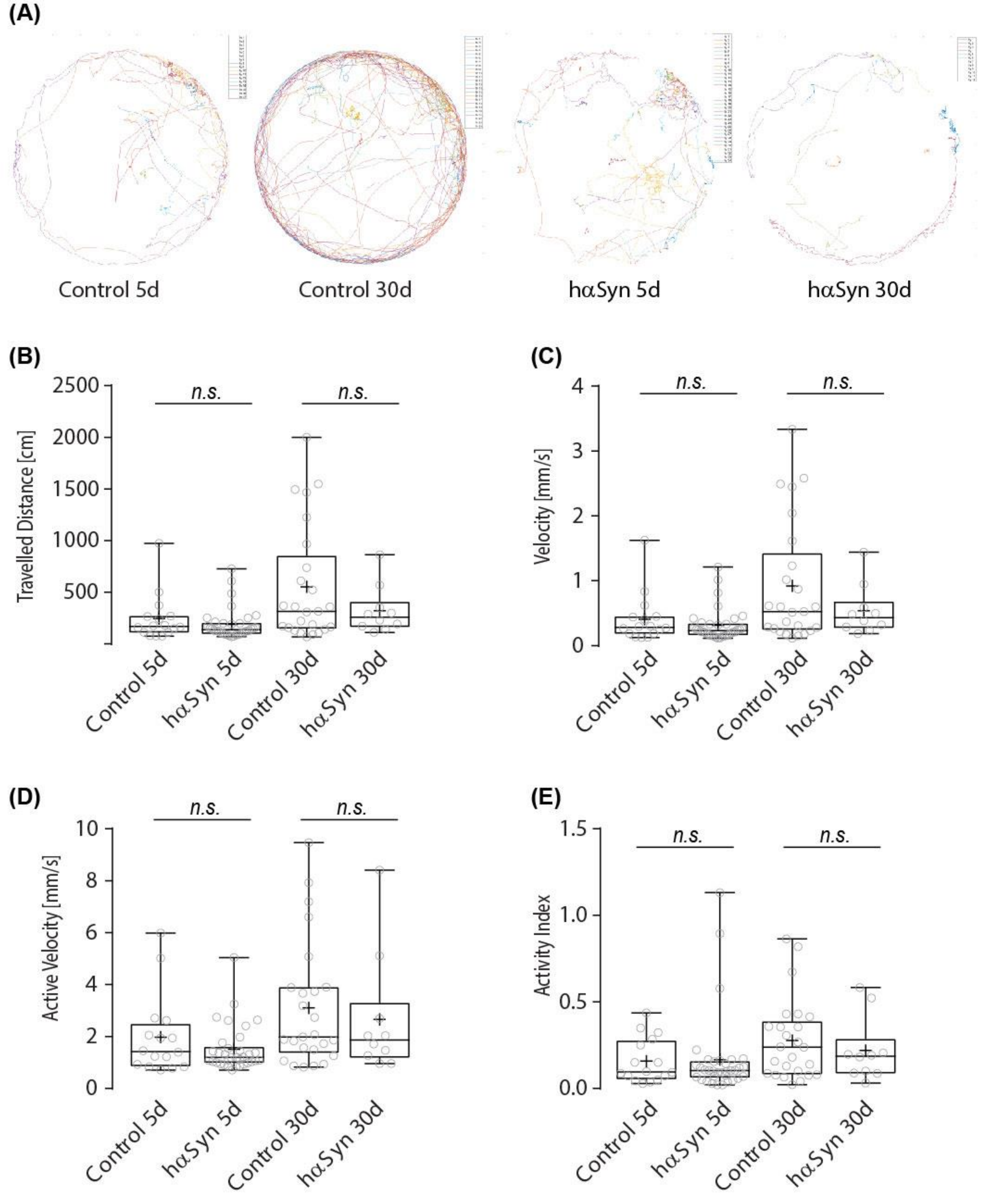


**Supplementary Figure S3: Spontaneous locomotor activity is not affected by hα-SynA30P expression under control of the SS56217 Split-Gal4 driver line. A)** Walking traces of 5-day and 30-day old control flies (left) and flies expressing hα-SynA30P in PPL201 and PPL204 (right). **B)** Flies expressing hα–SynA30P in SS56217-Gal4 (hαSyn) do not show significant alteration in the travelled distance compared to control flies neither in 5-day nor 30-day old animals. Same set of locomotion behavior were analyzed for but for Velocity **C)**, Active Velocity **D)** and Activity Index **E)**. (n.s. > 0.05, Kruskal-Wallis test with Dunn's multiple comparison.

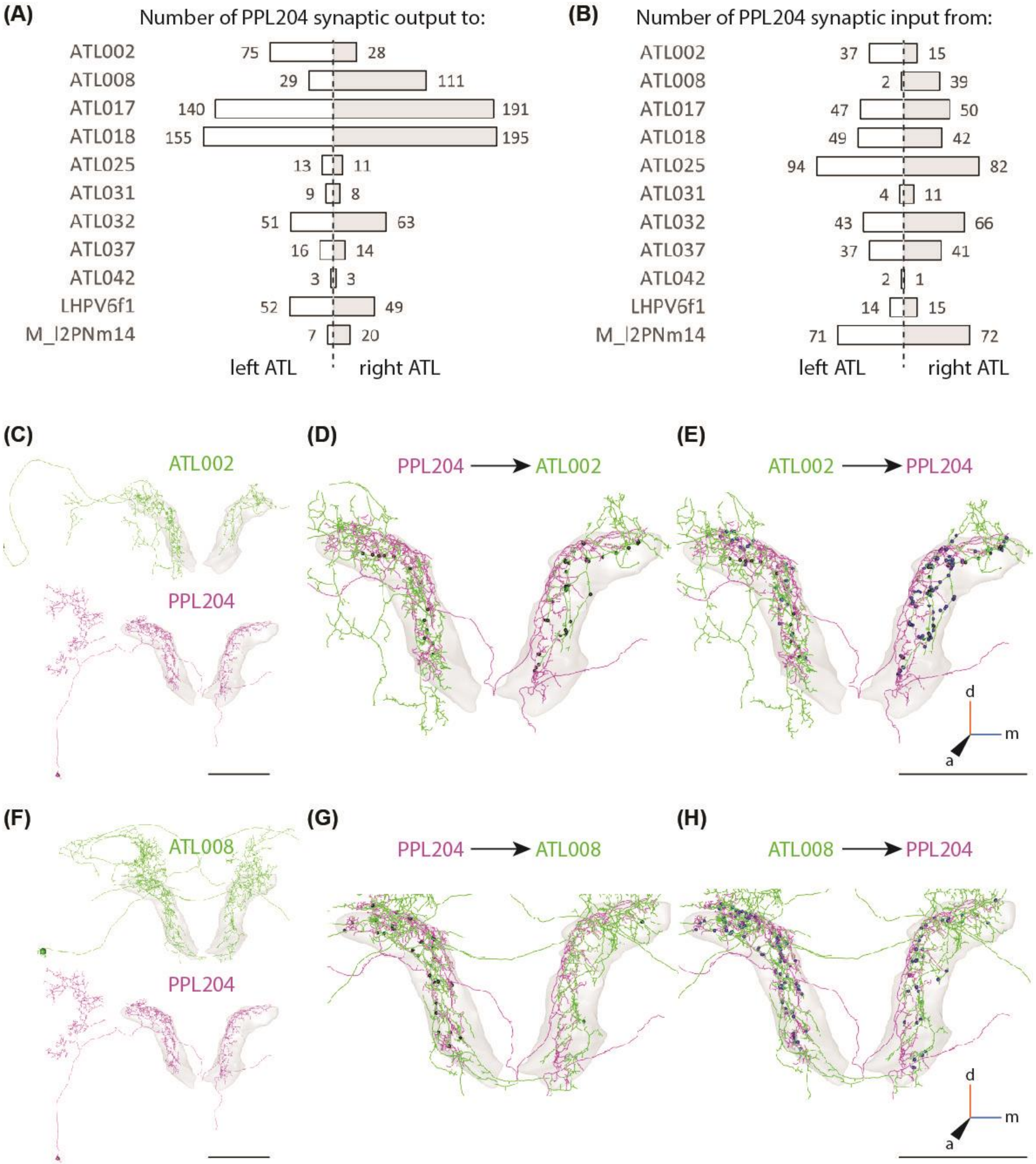


**Supplementary Figure S4: Pre- and postsynaptic connectivity between PPL204 DANs and interacting neurons in the ATL. A)** Number of PPL204 synaptic output target neurons in the left and right ATL neuropils. **B)** Number of PPL204 synaptic input from interacting neurons in the left and right ATL neuropils. **C)** ATL002, green and PPL204, magenta identified from FlyEM hemibrain data. **D)** PPL204 synaptic output (black dots) onto ATL002 in the left and right ATL neuropils. **E)** PPL204 synaptic input from ATL002 (black dots) in the left and right ATL neuropils. **F)** ATL008, green and PPL204, magenta identified from FlyEM hemibrain data. **G)** PPL204 synaptic output onto ATL008 (black dots) in the left and right ATL neuropils. **H)** PPL204 synaptic input from ATL008 (black dots) in the left and right ATL neuropils. Scale bars: 50 µm in C), E), F) and H).

*Supplementary Methods:*

To test for spontaneous horizontal locomotor activity in flies expressing UAS-h$\alpha$SynA30P pan-neuronally or in specific DANs 5- to 7-day-old and 29- to 31-day-old females were transferred to a circular free-walking arena (as described in [77]) of 120 mm diameter covered with a siliconizing reagent coated (Sigmacote; Sigma-Aldrich, Darmstadt, Germany) watch glass to prevent flies from walking on the covering glass. To allow for video recording without visual stimuli, the arena was illuminated by a ring of 60 concentrically arranged, mainly horizontally emitting infra-red LEDs (wavelength: 870 nm) and equipped with an infrared (IR)-sensitive high-speed camera (model VC-2MC-M340; Vieworks, Anyang, Republic of Korea) with a resolution set to 1760 by 1738 pixels and a frame rate of 20 frames per second (fps). IR LEDs were synchronized to the frame rate of acquisition. IR LEDs were synchronized to the frame rate of acquisition. To block all ambient visible or stray light and the light from stimulation LEDs, the camera's lens was equipped with IR-pass filter (cut-off frequency: 760 nm). All trials were carried out between 9 a.m. and 12 a.m., in consideration of the daily locomotor activity peak in the morning. After 5 min acclimatization time for each trial up to 8 female flies were recorded for 10 min to measure spontaneous activity simultaneously and 5 min for opto-genetic activation of neuronal subsets in the arena.

Videos were converted to grayscale, and a Look up Table (LUT) was inverted using FIJI (ImageJ 1.53c) to analyse the horizontal locomotion behaviour. Flies were tracked with the Caltech multi-platform Fly Tracker [78] and imported in MATLAB. Size of arena was set to 119 mm (1 mm smaller than the real arena) to exclude mirrored signal from the wall. Foreground and body thresholds were set for each video, so that all flies could be tracked through the whole video and walking threshold was set to 0.5 mm / s [79]. Velocity is defined as the average speed measured throughout the experiment; active velocity is defined as the average speed measured throughout the experiment excluding values measured below 0.5 mm / s. The Activity Index (AI) indicates the ratio between time spent walking and time spent not walking (grooming, standing, etc.), with higher values indicating that the flies spent more time walking than non-walking.

Tracking errors were manually corrected using the visualizer of FlyTracker and Excel. Few badly tracked flies (less than 5% of total tracked flies) were excluded from the results. The X and Y coordinates of each frame were generated by FlyTracker as Excel sheet. Traces were generated in MATLAB.

Walking distances, average velocities, average active velocities, and active indices were calculated in Excel. Bar plots were generated in Prism GraphPad. Pairwise T-tests were performed.
